# The International Text-Book of Surgery

**Published:** 1900-04

**Authors:** 


### BOOK REVIEWS, PAMPHLETS, EXCHANGES.

### BOOK REVIEWS.

[Contributions solicited for review.]

THE INTERNATIONAL TEXT-BOOK OF SURGERY. By American and British Authors. Edited by J. Collins Warren, M.D., LL.D., Professor of Surgery in Harvard Medical School, and A. Pearce Gould, M.S., F.R.C.S., Surgeon to Middlesex Hospital, Member of the Court of Examiners of the Royal College of Surgeons, England. Volume II. Regional Surgery. Published by W. B. Saunders, Philadelphia. Price, Cloth, $5.00 net; Sheep, $6.00 net.

This second volume on Regional Surgery will no doubt receive a hearty reception from the medical profession. This, too, is as it should be, for this last volume is not only filled with practical matter, but the various chapters are written by men nearly all of whom are eminently fitted for the task. There are some chapters which show marks of unusual preparation, both in the text and in the accompanying illustrations. Mention might be made of the excellent chapter on *Surgery of the Neck*, by A. Pearce Gould, M.S., F.R.C.S., which is unusually clear, concise and complete. The illustrations are good and well adapted. The chapter on *Surgery of the Breast*, by Dr. J. Collins Warren, is one of the best and most complete in the whole volume. The illustrations are works of art, containing several chromolithographs. The chapter by Dr. Chas McBurney, of New York, upon the *Surgery of the Vermiform Appendix* also needs special mention. Drs. F. Henrotin and M. L. Harris, of Chicago, have occupied two chapters on Gynecology, and these two chapters, while being well written, contain also numerous excellent illustrations.

The chapter on Surgery of the Ear is written by Dr. J. Orne Green in his excellent style, and although concise, he is very clear on his text. The chapters on the Surgery of the Nose and Eyes, while very good, are not so excellent as some other portions of the book, and the chapter by Mr. Treacher Collins on the Eye contains no illustrations except one or two pathological specimens. Altogether the work is one which can be used for ready reference, and always with a feeling of security as to its teachings. R.

